# An *ABCC9* Missense Variant Is Associated with Sudden Cardiac Death and Dilated Cardiomyopathy in Juvenile Dogs

**DOI:** 10.3390/genes14050988

**Published:** 2023-04-27

**Authors:** Eva Furrow, Nicole Tate, Katie Minor, Shannon Martinson, Shannon Larrabee, Marjukka Anttila, Meg Sleeper, Paula Henthorn

**Affiliations:** 1 College of Veterinary Medicine, University of Minnesota, St. Paul, MN 55455, USA; 2 Atlantic Veterinary College, University of Prince Edward Island, Charlottetown, PE CIA 4P3, Canada; 3 Pathology, Finnish Food Authority, 00790 Helsinki, Finland; 4 College of Veterinary Medicine, University of Florida, Gainesville, FL 32610, USA; 5 School of Veterinary Medicine, University of Pennsylvania, Philadelphia, PA 19104, USA

**Keywords:** channelopathy, canine, sudden cardiac death in the young, molecular autopsy

## Abstract

Sudden cardiac death in the young (SCDY) is a devastating event that often has an underlying genetic basis. Manchester Terrier dogs offer a naturally occurring model of SCDY, with sudden death of puppies as the manifestation of an inherited dilated cardiomyopathy (DCM). We performed a genome-wide association study for SCDY/DCM in Manchester Terrier dogs and identified a susceptibility locus harboring the cardiac ATP-sensitive potassium channel gene *ABCC9*. Sanger sequencing revealed an *ABCC9* p.R1186Q variant present in a homozygous state in all SCDY/DCM-affected dogs (*n* = 26). None of the controls genotyped (*n* = 398) were homozygous for the variant, but 69 were heterozygous carriers, consistent with autosomal recessive inheritance with complete penetrance (*p* = 4 × 10^−42^ for the association of homozygosity for *ABCC9* p.R1186Q with SCDY/DCM). This variant exists at low frequency in human populations (rs776973456) with clinical significance previously deemed uncertain. The results of this study further the evidence that *ABCC9* is a susceptibility gene for SCDY/DCM and highlight the potential application of dog models to predict the clinical significance of human variants.

## 1. Introduction

Molecular autopsies for sudden cardiac death in the young (SCDY) reveal pathogenic or likely pathogenic variants in 6–29% of cases [1,2,3,4,5]. The rate of molecular diagnosis of dilated cardiomyopathy (DCM) is similar [6]. Identification of susceptibility variants for SCDY and DCM increases diagnostic rates and enables screening of family members. However, validation of candidate genes and prediction of pathogenicity for specific variants is complicated by inability to perform segregation analyses in most families, due to only one or few affected individuals [3,4,7,8,9]. As such, many of the candidate genes for SCDY and DCM lack strong evidence of disease association [9,10,11]. Canine models offer unique advantages for validating disease-associated genes and variants. Due to population bottlenecks from limited founders and selective breeding, individual dog breeds have relatively little genetic diversity with disease traits often controlled by a small number of pathogenic variants [12]. This population structure is advantageous for the identification of disease-associated genes and variants. Dogs share susceptibility genes for many inherited diseases with humans, including sudden cardiac death and DCM [13,14,15,16]. Research in dogs has also identified novel susceptibility genes for sudden cardiac death and DCM [17,18,19]. Thus, canine models can be used to better understand molecular contributions to SCDY/DCM in humans and inform development and interpretation of molecular gene panels.

The Manchester Terrier provides a naturally occurring animal model of SCDY/DCM with disease manifesting as sudden death before 2 years of age, typically by 6 months [20]. Necropsy findings support acute and chronic forms. In the acute form, the heart is macroscopically normal with histopathologic abnormalities of acute multifocal myocardial degeneration and necrosis without inflammation. In the chronic form, mild cardiomegaly, left ventricle dilation, left ventricular wall thickening, and left auricle enlargement are common; in addition to myocardial degeneration, histopathologic abnormalities include myocardial fibrosis, mild inflammation, and, less frequently, myocardial mineralization. Dogs appear healthy prior to sudden death, with reports of anesthetic events or exercise preceding death in some cases [20].

In this study, we sought to discover the susceptibility gene for SCDY/DCM in Manchester Terrier dogs. We report results of a genome-wide association study (GWAS), sequencing of positional candidate genes, and follow-up genotyping of a putative causal variant for SCDY/DCM.

## 2. Materials and Methods

### 2.1. Study Population

**Figure 1:**
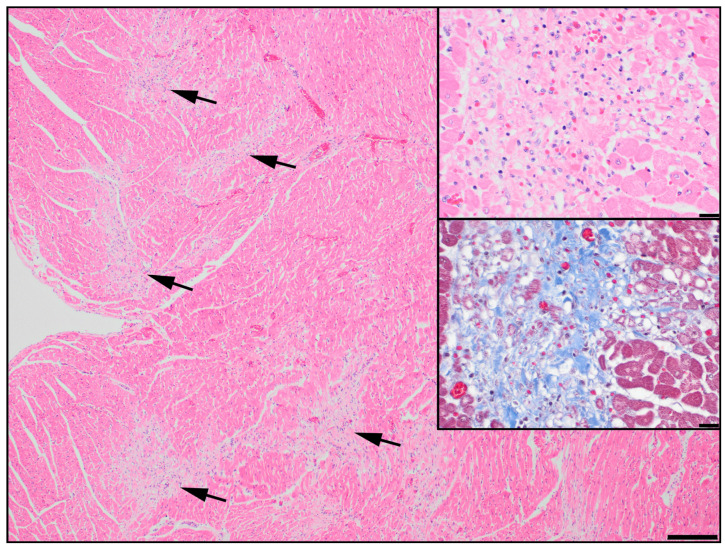
Right ventricle from a juvenile Manchester Terrier showing patchy regions of pallor in the myocardium (arrows). HE, scale = 200 µm. Upper inset: In these areas, there is myofiber degeneration and loss with replacement by connective tissue and scant mononuclear cells. Degenerate myofibers are present in the upper right half of the inset and are small (attenuated) with flocculent cytoplasm. Normal myofibers are evident at the lower right corner for comparison. HE, scale = 20 µm. Lower inset: Staining with Masson’s trichrome demonstrates collagen deposition (blue) in the affected regions. Scale = 20 µm.

Purebred Manchester Terrier dogs were enrolled in the initial study cohort, used for the GWAS. This GWAS cohort comprised 48 Manchester Terriers, including 12 cases and 36 controls. The 12 cases were dogs that passed away suddenly before 2 years of age and were confirmed to have DCM by postmortem histopathology characterized by foci of myocardial degeneration with fibrosis and mild lymphoplasmacytic infiltrates (Figure 1); 10 of the 12 dogs were included in a previous study on the histological characterization of the disease [20]. The average age at the time of death was 6 ± 4 months (range 2–14 months). The case population included seven female dogs and five male dogs; four of the five male dogs were reported to be unilaterally or bilaterally cryptorchid, and the reproductive status for the fifth male was not reported. The GWAS controls comprised 36 dogs of at least 2 years of age (average 8.0 ± 4.1 years, range 2–15 years) and unrelated within two generations; the controls included 20 females and 16 males, none of which were cryptorchid.

**Figure 2:**
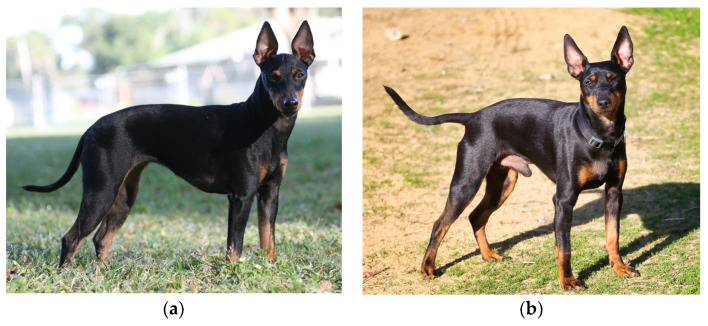
Photographs of two closely related dog breeds affected by sudden cardiac death of the young and dilated cardiomyopathy: (**a**) Manchester Terrier dog; (**b**) English Toy Terrier dog.

Additional dogs were included in follow up genotyping to validate the association of a variant of interest with SCDY/DCM. Both Manchester Terriers and English Toy Terriers were recruited during this study phase. These are closely related breeds with similar appearances (Figure 2), and pedigrees for some study dogs contained both breeds. The final study population comprised 19 cases with SCDY with or without pathologic confirmation of DCM (15 Manchester Terriers and 4 English Toy Terriers), 398 controls ≥2 years old without known heart disease (225 Manchester Terriers and 173 English Toy Terriers), 401 unknowns <2 years old without known heart disease (194 Manchester Terriers and 207 English Toy Terriers), and 3 with other cardiac disease (English Toy Terriers with hypertrophic cardiomyopathy). Dogs in the unknown phenotype group were followed into adulthood (2 years of age or beyond) to determine their outcomes, and 7 additional dogs were diagnosed with SCDY/DCM (total of 26 cases). The study protocol was approved by the University of Minnesota Institutional Animal Care and Use Committee (protocol ID 1509-33019A), and written informed consent was obtained from dog owners. Paraffin-embedded tissue (for deceased dogs), whole blood (3–4 mL in an EDTA tube), or cheek swab samples were collected from each dog. DNA was extracted using the Puregene Blood Kit (Qiagen) following manufacturer instructions. Extracted DNA was stored at −20 °C prior to genotyping.

### 2.2. SNP Genotyping and Analysis

SNP genotyping was performed with the Illumina CanineHD BeadChip (173,931 SNPs). Genotype quality control measures were performed using PLINK v1.90b3.30 (RRID SCR_001757) [21], a whole genome analysis toolset, and included exclusion of SNPs with genotyping calls <90% (11,937 SNPs removed), SNPs with a minor allele frequency <5% (68,520 SNPs removed), SNPs with differential missingness between cases and controls at *p* ≤ 0.01 (1297 SNPs removed), and SNPs that reject the hypothesis of Hardy–Weinberg equilibrium in the control population at *p* ≤ 0.001 (218 SNPs removed). The final dataset of 91,959 SNPs was analyzed with a linear mixed model implemented in GEMMA version 0.94.1 to control for population stratification [22]. *p*-values were calculated with the Wald test. R software for statistical computing was used to generate plots (R version 4.1.0, http://www.R-project.org/, accessed on 10 June 2021). Population stratification was assessed by calculating the genomic inflation factor from the *p*-value distribution.

### 2.3. Haplotype Analysis and Sequencing

Haplotypes for the region encompassing the top SNPs (29 with *p* < 10^−8^) were inferred from the SNP genotype data with the fastPHASE program version 1.2.3 [23]. The critical region was defined by where all 12 cases from the GWAS were homozygous for a shared haplotype. One of the cases was selected for Sanger sequencing of two positional candidate genes, *ABCC9* and *KCNJ8* (primers provided in Table S1).

### 2.4. Variant Selection, Genotyping, and Association with SCDY/DCM

Genomic variant locations were determined through a BLAT search (RRID SCR_011919) of the Dog10K_Boxer_Tasha/canFam6 assembly (GenBank assembly accession GCA_000002285.4) for 35–45 bases of the variant-containing sequence [24,25]. Transcript ENSCAFT00000043641.4 and UniProt J9NYX2 were used to determine the variant position and effect on the protein sequence for *ABCC9,* and ENSCAFT00000049622.3 and UniProt J9NU52 were used for *KCNJ8*. If the variant was a known single nucleotide polymorphism in dogs, the allele frequency was determined based on data in dbSNP *Canis lupus familiaris* build 151 [26]. Variant allele frequency was also determined in the Dog Biomedical Variant Database Consortium database of whole genome sequencing variant calls from 813 dogs (137 breeds and 9 wolves) [27].

Two in silico programs, PolyPhen-2 (RRID SCR_013189) and MutPred2 (RRID SCR_010778), were used to predict pathogenicity for missense variants [28,29]. For PolyPhen-2, the HumVar model was used because it is recommended for Mendelian diseases [28]. The University of California Santa Cruz Genome Browser was used to convert variant positions in the canine assembly to the position for the homologous nucleotide in the human assembly GRCh38/hg38 [30]. Nucleotide conservation scores were determined for variants using the “100 Vertebrates Basewise Conservation by phyloP (phyloP100way)” track on the UCSC Genome Browser [31]. PhyloP scores are the –log10 (*p* value) for rejecting the null hypothesis of neutral evolution; positive scores indicate conservation. The frequency of homologous human variants was determined using dbSNP *Homo sapiens* build 155. The AlphaFold Protein Structure Database was searched to identify the ABCC9 predicted protein structure [32,33]. The human UniProt entry O60706-F1 was used for structure prediction because the only canine UniProt entry with a prediction, A0A5F4DDH2-F1, was incomplete with only 149 of the 1549 amino acids. PyMOL Version 2.5.5 (RRID SCR_000305) was used for visualizing the location of an *ABCC9* missense variant [34], and Missense3D was used to predict structural changes using the AlphaFold model [35].

The putative pathogenic variant was genotyped in the full cohort of 821 dogs using Sanger sequencing of PCR products spanning the variants (Table S1) or custom TaqMan SNP Genotyping Assay (Assay ID: ANAAPHA, Thermo Fisher Scientific Inc., Waltham, MA, USA). A chi-square test was used to test the association between SCDY/DCM and a homozygous genotype for the putative pathogenic variant.

## 3. Results

### 3.1. Discovery of an ABCC9 Missense Variant in a Natural Canine Model of SCDY/DCM

**Figure 3:**
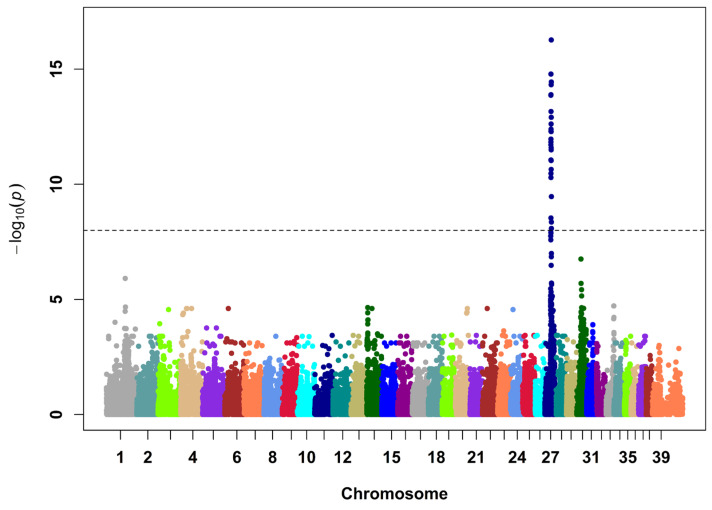
Manhattan plot of a mixed model genome-wide association study for sudden cardiac death in the young and dilated cardiomyopathy in 12-case and 36-control Manchester Terrier dogs. There is a strong signal on chromosome 27 (*p* = 5 × 10^−17^ Wald test) at a locus that harbors genes encoding subunits of an ATP-sensitive cardiac potassium channel (*ABCC9* and *KCNJ8*). Single nucleotide polymorphisms above the black dotted line achieved a *p* value < 10^−8^.

The GWAS of 48 Manchester Terrier dogs (12 SCDY/DCM cases and 36 controls) revealed a strong signal on CFA27 (*p* = 5 × 10^−17^, genomic inflation factor = 1.2; Figure 3, Figures S1 and S2, and Table S2). The most strongly associated haplotype spanned 1.9 Mb (CFA27 g.24710989–26639517 bp, Broad CanFam3.1/canFam3; CFA27 g.19817148–21651279 bp, Dog10K_Boxer_Tasha/canFam6) and contains 25 protein-coding genes (Table S3). All cases and no controls were homozygous for the risk haplotype in this region.

**Figure 4:**
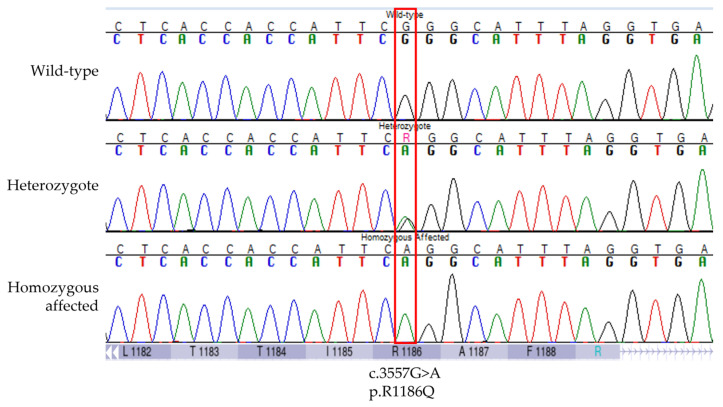
Representative Sanger sequencing electropherograms for wild-type, heterozygous, and homozygous affected genotypes for the *ABCC9* p.R1186Q variant (ENSCAFT00000043641.4 c.3557G > A) associated with sudden cardiac death in the young and dilated cardiomyopathy in a natural dog model.

Of the positional genes, *ABCC9* and *KCNJ8* were the top candidates based on ontology and previous evidence for a role in inherited cardiac disorders. *ABCC9* and *KCNJ8* encode subunits of cardiac ATP-sensitive potassium (K_ATP_) channels, SUR2 and Kir6.1, respectively [36,37]. These genes have been implicated in DCM, Cantú syndrome, atrial fibrillation, Brugada (J-wave) syndrome, and sudden death in infants and adults [7,8,38,39,40,41,42,43,44,45,46,47]. Exonic sequencing of *ABCC9* and *KCNJ8* in an affected dog revealed two *ABCC9* variants that were absent from controls (Table 1). One was a synonymous variant, *ABCC9* p.D1316=, that is a common SNP in dogs (rs852067132). The other was a missense variant, *ABCC9* p.R1186Q, that alters a highly conserved residue within the ABC transmembrane domain 2 (TMD2) and is predicted to be deleterious by in silico analyses (Figure 4 and Figure S3, and Table 1). Missense3D predicted two structural changes as a result of this variant: buried charge replaced (replacement of a buried charged residue with an uncharged residue) and cavity altered (expansion of the cavity volume by 80 Å^3^). The p.R1186Q variant was not present in dbSNP *C. lupus familiaris* build 151 or the Dog Biomedical Variant Database Consortium database.

### 3.2. Validation of the Association between ABCC9 p.R1186Q and SCDY/DCM

Additional dogs were recruited to determine population frequency and penetrance of *ABCC9* p.R1186Q in Manchester Terriers and a closely related breed, the English Toy Terrier, that is also affected by SCDY/DCM. Eight hundred and twenty-one dogs were genotyped, including 19 SCDY/DCM cases, 398 adult controls, 401 juvenile dogs with unknown phenotypes, and 3 adult dogs with hypertrophic cardiomyopathy. The variant was present in a homozygous state (100% allele frequency) in all SCDY/DCM cases. No control was homozygous for *ABCC9* p.R1186Q, but 69 heterozygous carriers were detected with a variant allele frequency of 0.08 in Manchester Terriers and 0.09 in English Toy Terriers. The three adult dogs (2, 6, and 8 years old) with hypertrophic cardiomyopathy were clear of the variant. The variant was present in a homozygous state in 9 of 401 juvenile dogs (<2 years of age) of undetermined phenotype. Three of these dogs died suddenly as puppies (i.e., SCDY, two at 7 weeks of age and one at 11 months), and four were euthanized (one at 2 months and three at 4 months of age); in these seven homozygous dogs, DCM was histologically confirmed on necropsy. The affected dog that was euthanized at 2 months of age underwent an echocardiogram and electrocardiogram shortly before death, and no abnormalities were detected. The two other homozygous dogs were lost to follow up; both dogs also reportedly had normal echocardiographic findings, one had a normal electrocardiogram, and the other had a brief run of sinus tachycardia (>300 bpm). In total, all 26 dogs with SCDY/DCM and none of the 398 control dogs were homozygous for *ABCC9* p.R1186Q (*p* = 4 × 10^−42^), consistent with autosomal recessive inheritance with complete penetrance. As previously reported [20], cryptorchidism was common in male dogs with SCDY/DCM, with 10 of 15 males homozygous for *ABCC9* p.R1186Q having unilateral or bilateral cryptorchidism. Of the five other homozygous males, three were too young (≤2 months of age) to determine if the testes were going to descend, and two did not have information provided on the status of the testes.

### 3.3. Homologous ABCC9 Variants in Human Databases

We next checked dbSNP *H. sapiens* build 155 to determine if a homologous variant exists in humans. *ABCC9* p.R1186Q is a rare variant in humans (rs776973456, allele frequency ≤ 0.0007), detected in American, European, and Korean populations. It has been previously reported in a heterozygous state in a human with restrictive cardiomyopathy that also had a heterozygous variant in *MHY7* (encoding β-myosin heavy chain) [48]. Another missense variant, p.R1186W (rs886049169), also exists at this residue in humans at lower frequency (allele frequency ≤ 0.000007). Both the p.R1186Q and p.R1186W variants are currently classified as having uncertain significance by ClinVar [49].

## 4. Discussion

Our results demonstrate that the p.R1186Q variant in the cardiac ATP-sensitive potassium channel subunit gene, *ABCC9*, is associated with SCDY/DCM in a natural canine model. Homozygosity for the p.R1186Q variant had complete penetrance for sudden death before two years of age. The current ClinGen clinical validity classification for the role of *ABCC9* in DCM is “limited” [11]. The striking disease association here strengthens the evidence for a causal role of *ABCC9* in SCDY and DCM.

The triple-risk model for sudden infant death syndrome includes three co-existing risk factors: (1) a vulnerable infant, (2) a critical developmental period, and (3) an exogenous stressor(s) [50]. Expression of *ABCC9* increases with the transition from fetal glycolytic metabolism to mitochondrial oxidative metabolism in the newborn heart [51]. Cardiomyocytes lacking functional ABCC9 have reduced fatty acid oxidation and oxygen consumption and are unable to respond to cell stress [51]. Thus, the discovery of an *ABCC9* variant that causes SCDY/DCM, with death potentially triggered by physiologically stressful events, is in alignment with the triple-risk model. It is unknown whether antiarrhythmic drugs or other therapies during the critical development stage might prevent SCDY in puppies homozygous for the *ABCC9* p.R1186Q variant. In a Kir6.2 (another subunit of the cardiac K_ATP_ channel) knockout mouse model, calcium-channel blockade with verapamil reduced sudden death [52]. In humans, genotype-targeted therapies are emerging for prevention of sudden cardiac death in patients with genetic risk factors [53].

The ABCC9 protein is a regulatory subunit of cardiac K_ATP_ channels and consists of two 6-helix transmembrane domains (TMD1 and TMD2), a 5-helix N-terminal domain (TMD0), and two nucleotide binding folds (NBF1 and NBF2) [54]. The SCDY/DCM associated p.R1186Q variant resides within TMD2. This variant was previously detected in a heterozygous state in a human with restrictive cardiomyopathy; however, the presence of a concurrent variant in *MYH7*, an established susceptibility gene for restrictive cardiomyopathy, complicated prediction of the role of the *ABCC9* variant in the cardiac phenotype of that patient [48,55]. A nearby rare variant in the TMD2, p.R1197C (rs778849288), has been identified in humans with sudden unexplained nocturnal death syndrome or DCM [7,47]. Another nearby variant, p.M1198I (rs199900459), was reported in a patient with left ventricular non-compaction cardiomyopathy [56]. The TMD2 is also a common site for gain of function variants causal for Cantú syndrome [42,43]. The pathogenicity of p.R1186Q is supported by the high conservation of the R1186 residue and its location in a hot spot for cardiac phenotypes. However, the absence of functional assays is a limitation of this study, and the specific effect of the variant on cardiac K_ATP_ channel function is unknown. The ABCC9 subunit of the cardiac K_ATP_ channel regulates its inhibition by ATP, and this inhibition is decreased by Cantú syndrome *ABCC9* variants, including those located in TMD2 [42]. Some of the *ABCC9* variants identified in human SCDY patients similarly exhibit reduced sensitivity to ATP inhibition [8]. The SCDY/DCM associated p.R1186Q variant identified here is predicted to cause structural changes to the protein, including replacement of a buried charge and expansion of cavity volume, which could alter ATP sensitivity or have other effects on channel function.

In addition to the cardiac phenotype, most affected male dogs were unilaterally or bilaterally cryptorchid. Cryptorchidism is not a reported phenotype of variants in *ABCC9* or the other genes residing in the risk haplotype [49]. However, hypertrophic cardiomyopathy and cryptorchidism are common features of RASopathies, such as LEOPARD syndrome, Noonan syndrome, Costello syndrome, and cardiofaciocutaneous syndrome [57], and pathway analysis of cryptorchidism candidate genes includes associations with “cardiac muscle contraction”, “dilated cardiomyopathy”, and “hypertrophic cardiomyopathy” [58]. Since only *ABCC9* and *KCNJ8* were selected for sequencing in this study, we cannot determine whether the reported *ABCC9* variant is the top candidate for cryptorchidism in the affected Manchester Terriers or if there is a more likely causal variant in a different gene that is present in linkage disequilibrium.

The present discovery adds to a growing list of shared susceptibility genes for sudden cardiac death and DCM between dogs and humans [59]. For example, variants in *KCNQ1* (potassium voltage-gated channel subfamily Q member 1) are one of the most common causes of long QT syndrome in humans, and a *KCNQ1* variant is associated with this syndrome in dogs [13,60]. The most common DCM susceptibility variant in humans, *TTN* (titin), is also a major underlying contributor to sudden cardiac death and DCM in dogs [6,14]. Other examples of shared susceptibility genes between these species include *PLN* (phospholamban), *RBM20* (RNA-binding motif protein 20), and *DMD* (dystrophin) [11,15,16,61,62,63]. In addition to strengthening pathogenicity evidence for known candidate genes in humans, studies in dogs have revealed novel susceptibility genes for sudden cardiac death, DCM, or both, such as *STRN* (striatin), *PDK4* (pyruvate dehydrogenase kinase 4), and *QIL1/MICOS13* (mitochondrial contact site and cristae organizing system subunit 13) [17,18,19]. The success of molecular autopsies for diagnosing genetic causes of sudden cardiac death depends on the comprehensiveness of the gene panel, the evidence level for the gene, and the ability to predict pathogenicity of specific variants. There are greater than 70 candidate genes for sudden cardiac death and greater than 40 for nonsyndromic DCM, but many lack definitive evidence to confirm their role in these diseases [10,11]. A study on results of panel testing of more than 200 genes in patients with inherited cardiomyopathies or channelopathies found that 61% of variants detected resided in genes with insufficient evidence to confirm disease association, and 70% of those were variants of unknown significance [64]. Data from dogs and other natural animal models of sudden cardiac death and DCM can contribute to evidence of gene associations with disease and variant pathogenicity and thereby hold value in improving the accuracy of molecular autopsies.

In conclusion, while the functional effect of the *ABCC9* p.R1186Q variant on cardiac K_ATP_ channels is undetermined, the results of this study strengthen the evidence that *ABCC9* is a susceptibility gene for sudden cardiac death and DCM in infants, children, and adults. The perfect correlation between homozygosity for p.R1186Q and SCDY/DCM in Manchester Terrier dogs adds to the in silico evidence that it is a pathogenic variant [65]. Genotype–phenotype association is often difficult to confirm for rare variants in humans. The results of this study demonstrate how spontaneous dog models can help validate disease associations for susceptibility variants and thus improve confidence in the clinical significance of homologous human variants. The discovery of the *ABCC9* p.R1186Q also enables genetic testing of breeding dogs to prevent affected puppies from being produced.

## Figures and Tables

**Table 1:** Exonic variants identified in *ABCC9* in a Manchester Terrier dog with sudden cardiac death in the young and dilated cardiomyopathy.

Genomic Alteration *	Protein Alteration †	PhyloP	PolyPhen-2 HumVar	Mut-Pred2	Allele Frequency	
					dbSNP ‡	DBVDC
CFA27: g.21042635C > T	p.R1186Q	7.8	0.998	0.722 §	0	0
CFA27: g.21025527G > A	p.D1316=	1.7	NA	NA	0.34	0.30

DBVDC, Dog Biomedical Variant Database Consortium. * Genomic positions are based on the Dog10K_Boxer_Tasha/canFam6 assembly (GenBank assembly accession GCA_000002285.4). † Protein positions are based on transcript ENSCAFT00000043641.4 and UniProt J9NYX2. ‡ *C. lupus familiaris* build 151. § MutPred2 predicted three molecular mechanisms, including loss of allosteric site at R1186 (probability 0.28, *p* = 0.0066) and altered DNA binding (probability 0.19, *p* = 0.03, and altered transmembrane protein (probability 0.09, *p* = 0.05).

## Data Availability

Data that support the findings of this study are either included in the published article and its supplementary information files or available from the corresponding author upon reasonable request.

## Supplementary Materials

The following supporting information can be downloaded at: https://www.mdpi.com/article/10.3390/genes14050988/s1, Figure S1: Q-Q plot of the GWAS results; Figure S2: Plot of GWAS results for CFA27; Table S1: Top SNPs (*p* < 10^−8^) associated with SCDY/DCM in a mixed model GWAS in Manchester Terrier dogs (12 cases and 36 controls); Table S2: Protein-coding genes residing in the critical region associated with SCDY/DCM in a mixed model GWAS in Manchester Terrier dogs; Table S3: PCR primers used for sequencing of ABCC9 and KCNJ8 coding exons. Figure S3: Location of the p.R1186Q variant in the predicted ABCC9 protein structure.
