# Supplementary material for: An ABCC9 Missense Variant Is Associated with Sudden Cardiac Death and Dilated Cardiomyopathy in Juvenile Dogs
Source: Genes (Basel). 2023 Apr 27;14(5):988. doi: 10.3390/genes14050988 (PMC10218235; doi:10.3390/genes14050988)
Supplement: Supplementary file 1 [file genes-14-00988-s001.zip › genes-2358694-supplementary.pdf]

Supplementary Material

An *ABCC9* Missense Variant is Associated with Sudden Cardiac Death and Dilated Cardiomyopathy in Juvenile Dogs

Figure S1. Q-Q plot of the GWAS results.

Quantile-quantile (Q-Q) plot of 91,959 expected versus observed log-transformed *P* values from a mixed model genome-wide association study analysis for sudden cardiac death in the young and dilated cardiomyopathy in 12 case and 36 control Manchester Terrier dogs.

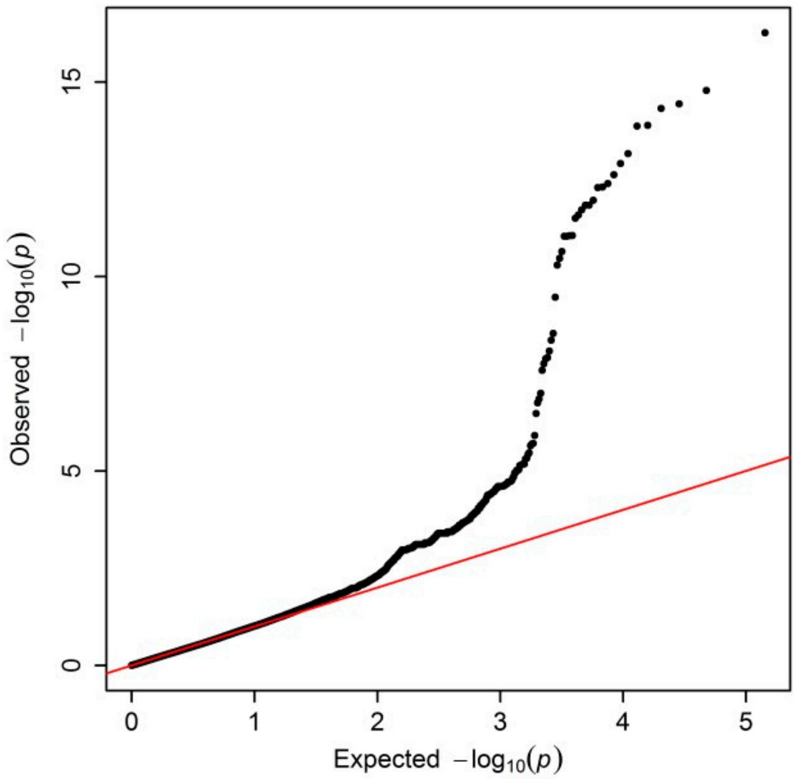

Figure S2. Plot of GWAS results for CFA27.

Plot of log-transformed *P* values for canine chromosome 27 SNPs (positions for Broad CanFam3.1/canFam3 GenBank GCA_000002285.2) from a mixed model GWAS analysis for sudden cardiac death in the young and dilated cardiomyopathy in 12 case and 36 control Manchester Terrier dogs. The region containing the top SNPs ($P < 10^{-8}$, highlighted in red) is bordered with dashed lines.

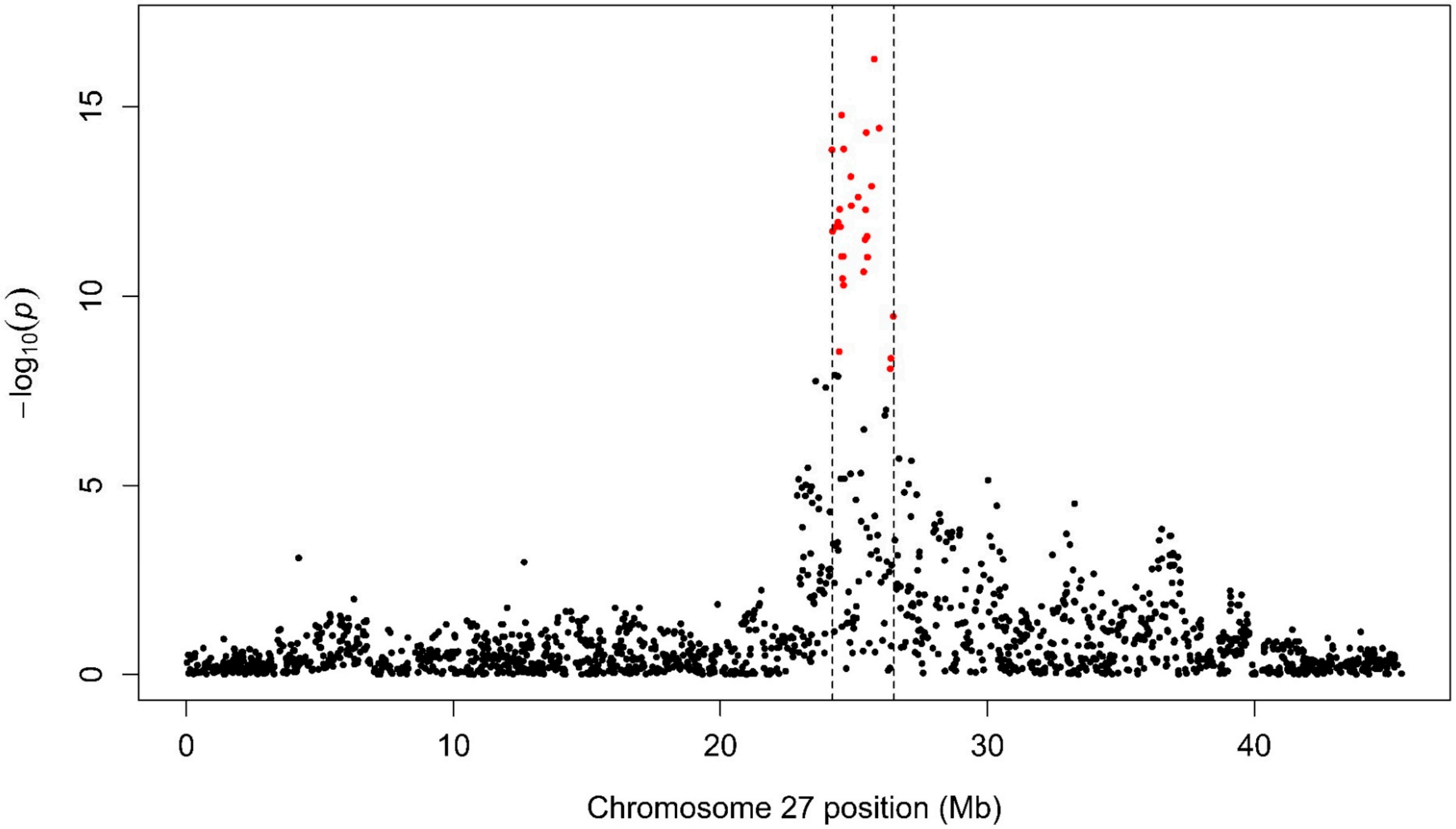

Figure S3. Location of the p.R1186Q variant in the predicted ABCC9 protein structure.

The human ABCC9 predicted protein structure (O60706-F1) was obtained from AlphaFold Protein Structure, and the image was created using PyMOL.

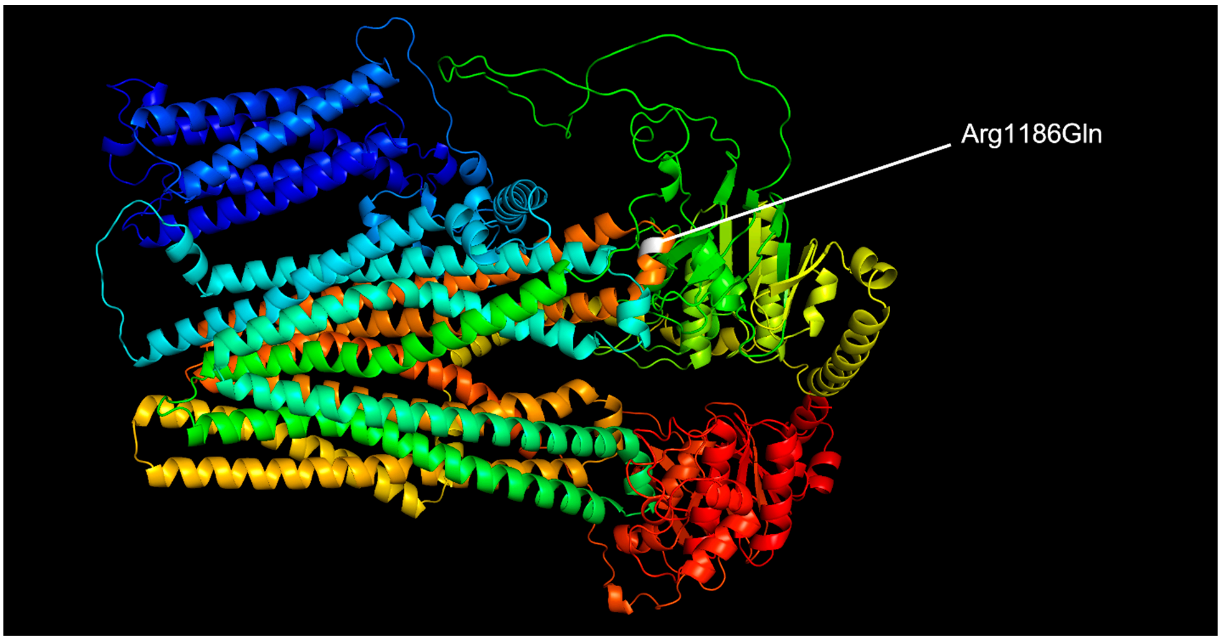

Table S1. Top SNPs ($P < 10^{-8}$) associated with SCDY/DCM in a mixed model GWAS in Manchester Terrier dogs (12 cases and 36 controls).

SNP	CHR	Position[a]	*P*
BICF2G630150840	27	25760276	5.46E-17
BICF2G630150101	27	24539912	1.65E-15
BICF2G630151366	27	25946871	3.66E-15
BICF2P327797	27	25462907	4.79E-15
BICF2G630150151	27	24618620	1.30E-14
BICF2S23019839	27	24177413	1.37E-14
BICF2P275003	27	24886868	6.96E-14
TIGRP2P351644_rs9069935	27	25664401	1.25E-13
BICF2P292584	27	25158323	2.42E-13
BICF2P908494	27	24900628	4.09E-13
BICF2P544057	27	24466539	5.06E-13
BICF2G630150445	27	25436543	5.22E-13
BICF2G630149956	27	24404459	1.10E-12
BICF2S23755936	27	24328638	1.47E-12
BICF2G630150066	27	24497260	1.47E-12
BICF2S23325539	27	24199526	1.94E-12
BICF2G630150523	27	25493817	2.62E-12
BICF2G630150430	27	25420527	3.21E-12
BICF2G630150142	27	24601913	8.87E-12
BICF2G630150093	27	24534441	8.99E-12
BICF2G630150531	27	25502241	9.31E-12
BICF2G630150563	27	25514727	9.31E-12
BICF2G630150344	27	25366550	2.28E-11
BICF2G630150129	27	24575466	3.43E-11
BICF2G630150147	27	24613771	5.10E-11
BICF2P1208930	27	26474738	3.43E-10
BICF2G630150035	27	24447332	2.91E-09
BICF2G630151587	27	26387601	4.38E-09
BICF2S23236113	27	26363778	8.31E-09

[a]Genomic positions are based on the Broad CanFam3.1/canFam3 assembly (GenBank GCA_000002285.2).

Table S2. Protein-coding genes residing in the critical region associated with SCDY/DCM in a mixed model GWAS in Manchester Terrier dogs. Genes are based on annotation for the region spanning CFA27 g.19817148-21651279 bp in the Dog10K_Boxer_Tasha/canFam6 assembly (GenBank GCA_000002285.4).

PDE3A	*PYROXD1*	*ENSCAFG00000024414*	*GOLT1B*	*ABCC9*
SLCO1C1	*ENSCAFG00000013746*	*ENSCAFG00000029531*	*SPX*	*ENSCAFG00000011854*
SLCO1B3	*ENSCAFG00000025484*	*ENSCAFG00000016289*	*GYS2*	*CMAS*
SLCO1A2	*ENSCAFG00000055974*	*ENSCAFG00000016278*	*LDHB*	*ST8SIA1*
IAPP	*ENSCAFG00000012292*	*RECQL*	*KCNJ8*	*C2CD5*

Table S3. PCR primers used for sequencing of *ABCC9* and *KCNJ8* coding exons. The primer set for the PCR product spanning the putative pathogenic variant is bolded.

Gene/Exon	Forward	Reverse	Product size, bp
ABCC9[a]			
4	TCTCAAATGACTTTGGCATACAG	GCACAGAAAACTGGCTTGTTC	479
5	TGGGATTTTCATTGCTACCG	TGCTATTAAATGCGTGACAAATG	432
6	CCCAACGTAGTCTTCCTTTGC	GGGGAAGAGAGTGCTCACAG	461
7	AACTCCAAAAGTGCATGAAGC	GGAACACTAATGGCCTGAAAC	510
8	GTGGATTAAACATGAAATTCTGG	TGTTCCTCCTATCCCTCCTC	704
9	TGCATGGAATGCAAGGATATAG	TTTCTGAACCTGGCTTCTGG	625
10	CCAAAATTGCCTTGCTATGG	TTCAGTGGGTTTACTAATTAGCTCTG	477
11	TGCTAAAATGAAGATAGTGGTTGAC	TTCCAAACCCCTTTCTTAACC	492
12	TCCAACAGTTCTTAGTTGAGTTGAC	TGTTTTTGCAACACAGAAAATACAC	446
13	AGAGCCAGGCAGAAATCAAG	TAGGCTCATGTGGCTAATGG	464
14	CAACTGGGCAATGTATGTGG	CCTTTGAGAAACCCTGGATTAG	371
15	ACAAGTTTACTGCGGGGATG	TCCCAGTGGTGACAAACAAG	573
16	TCAGAAAGGAAGGCCACTTG	GAACATGTGCTCAAAGAATTGG	482
17	TTTGGAGAGGCCATTGAAAG	CGGAACACTTTTGGAAATGC	421
18	TTAGAATTTCACCACTGTGTGTTC	GTGAGCTGCATGGTGCAAG	359
18	TTGTGTATCAGAGAAAGAAGTTATGC	AAATTTATGACATTTAAGGTTTCCAC	426
19	GGGTAGAACCCAGGTCACTG	TAAATGTTGGGAACCACTGC	487
20	TCAAATTGCACATTGGAAAAC	TAACCCTCTGCACCCTCCTC	472
21	TTTAAACACTTCTTTGGCATAGC	GACCACTTTCCTTCTGCTCCT	552
22	TGTCTTTGTATCCACAAACCTACTG	TCCTATGCTCCCATTTCTGG	390
23	GGAGGGCACTTGATTTGATG	TGATCCTCTGGTGTTTGCAC	460
24	GTGTCCCTGCAGAGTGTGG	TGGCTATGATCTAAGGAAAGCAC	437
25	CACATGCTGACTGGGTAAGAG	TTACTTTGGGTTTCAAATGAGG	427
26	CCTCAGACATTGCCTCACAAC	TTGCACACACCTTTCCTTTG	464
27	TGCTCATTCATTCATTTTCACC	GCGATCACATAGATTCCTTTTTG	607
28	GGAAAACTGCACTCCTTCAAC	TCTGCGCACTGCTAATGAAC	474
28	CAGCTGCCAAAAACCTTCAC	GCCCCTTCCTTTAAGTTTTTATTC	529
29	TGGGAAGTGAGTGAAGTTAAAGG	ATGCCCATGGCTATGTAAGG	542
**30**	**TCATGTTTCTCTTCCTTTTTAGGTC**	**CCCAATATGGTGTTTCAATCTTC**	**476**
31	CCTCTGCTTTGCCACTTCTC	CCCAATATGGTGTTTCAATCTTC	278
32	CGCACCAAAGCAGTAGTAGC	TTTTTGGGCTATTTGTCTTGC	482
33	TTCCACATCAGGCTCCCTAC	TCCACTCTGCCCTAAAATGC	431
34	CCATGACACTCTCCCTGGTC	GGCCTAGTCATATCGTTTCAGG	457
35	TCCCCTTTTCTCTCCCTCTC	TGGGAGACTAATGAACAGCAATAC	484
36	GGAGACCTAAGAAAAGAATGAGG	CTACAGGGAGCCTGATGTGG	372
37	GAGATGCATAATGATGTTTGTAACC	TTTCCCAAACCAAATTGTTG	412
38	ATGCCTGCTCTATCCTGAGC	CATTCTCACGGGAATGTGC	581
39	GGATGGCTCAGGATCATCTTC	TCTGAATGTGCTACCATCAGTTG	477
40	CACCTACACAGAAGGCAGGAG	AAGGCTTTCAGAGCATAGCTG	370
41	CCCAGTCACACACACTCACC	TGAAGCAGTAAAATCTTGGAAAAC	537
KCNJ8[b]			
3	GACATGGGGGAGGAGAAAG	CGAAGGCACTTAAAGCAAATG	613
4	CCTTTGGAGGGAGAAAATGG	TGCTTGAATATGAGCATCTGG	1100
4	AGAACCGTTTCCCAGAAGTC	CTTCTTGACCACCTGGATGC	549
4	GTGGGGGACCTAAGGAAGAG	TCGGATGGAGTTGTTTCTCC	564
4	CCCTCCAGAAGAGTGAGCTG	CAGAAAGTGCTGTTGCTTGA	288

[a]Exon number based on transcript ENSCAFT00000043641.4.
[b]Exon number based on transcript ENSCAFT00000049622.3.
